# Seasonal Prevalence of Gastrointestinal Parasites in Macaques (*Macaca thibetana*) at Mount Emei Scenic Area in China

**DOI:** 10.3390/ani12141816

**Published:** 2022-07-15

**Authors:** Jiandong Yang, Samuel Kumi Okyere, Jie Zheng, Buyuan Cao, Yanchun Hu

**Affiliations:** 1 College of Animal Sciences and Technology, Sichuan Agricultural University, Chengdu 611130, China; yangjd@sicau.edu.cn; 2 Key Laboratory of Animal Disease and Human Health of Sichuan Province, College of Veterinary Medicine, Sichuan Agricultural University, Chengdu 611130, China; samuel20okyere@gmail.com (S.K.O.); cao459108027@163.com (B.C.); 3 Forestry Management Agency of Mount Meishan, Meishan 614200, China; zhengjienew@126.com

**Keywords:** macaque, parasite, prevalence rate, *Gongylonema* spp., *Entamoeba* spp.

## Abstract

**Simple Summary:**

Gastrointestinal parasites may affect the health of macaques directly or indirectly, thereby exposing primates to conservational risks. Gastrointestinal parasites cause various health conditions such as apathy, diarrhea, malaise and weight loss. However, there is still a dearth of knowledge about the population of intestinal parasites in the macaques kept in the Mount Emei scenic spot. Therefore, using the microscopic detection method, the population of gastrointestinal parasites in fecal samples of monkeys at the Mount Emei scenic spot was estimated. The results showed that *Gongylonema* spp. and *Entamoeba* spp. were dominant gastrointestinal parasites in the fecal samples of monkeys at the Mount Emei scenic spot. This is the first reported study on gastrointestinal parasites in monkeys at the Mount Emei Scenic and will help in designing a future road map for parasitic disease monitoring and control in wild-life habitats, as well as provide epidemiological data on parasites in monkeys in the Mt. Emei Scenic Area at Sichuan, China.

**Abstract:**

The aim of the study was to elucidate the prevalence of intestinal parasites in macaques at the Mt. Emei Scenic Area of Sichuan, China. A total of 168 fecal samples were collected from yellow (*n* = 31), black (*n* = 19), new (*n* = 57), Leidongping (*n* = 57) and Wuxiangang (*n* = 4) macaques from 2019 to 2020. The fecal samples were tested for various gastrointestinal parasites following the microscopic detection method. The results showed that the total prevalence rate of the intestinal parasite was 51.19% (86/168), whereas the intestinal parasite with the highest prevalence was *Gongylonema* spp. (26.79%) for helminth and *Entamoeba* spp. (18.45%) for protozoa. Interestingly, the highest prevalence of intestinal parasites was observed during the summer season (86.21%), and the lowest was observed during the winter season (7.14%). There was a positive correlation observed between the human contact frequency and total prevalence rate of the intestinal parasites (*p* < 0.05); however, there was no correlation between the human contact frequency and total prevalence of the intestinal parasites at different seasons (*p* > 0.05). In conclusion, the dominant parasites *Gongylonema* spp. and *Entamoeba* spp. cause various diseases that may be transmitted to humans and other animals; therefore, there is a need for a proper management system, such as parasite control measures and population protection in the Mt. Emei Scenic Area of Sichuan, China.

## 1. Introduction

Knowledge of gastrointestinal tract (GIT) parasites provides important information for the management of wild animals in scenic areas, as these parasites pose a threat to the population of other wild and domestic animals, as well as tourists [1]. Gastrointestinal parasites reside in the small intestine (duodenum, ileum, jejunum) and large intestine (caecum, colon and rectum) [2]. Gastrointestinal parasites release their infectious propagules in the fecal matter of their hosts, which, when ejected, results in the buildup of infectious agents in the habitat, thereby increasing their transmission [3]. Infections by gastrointestinal parasites, including helminth and protozoans, have been reported in wild animals [4]. As a national second-class protected animal, macaques in the Mount Emei Tibetan area are a unique monkey species in China [5]. Macaques belong to the family of Mammalia and order Primate [6]. Macaques are non-human primates (NHP) mostly found in Africa, Asia and America [7,8]. Rhesus macaque share many common structural, physiology, immunity and heredity traits with humans [9]. For example, the homology of the nucleic acid sequence between macaques and humans was reported to be more than 95% [10]; hence, rhesus monkeys are important experimental animals in scientific research [11,12,13]. In recent years, the passenger flow at the Mount Emei scenic spot has increased rapidly. Moreover, macaques in the Mount Emei scenic spot have frequent contact with tourists who are allowed to take photos and play with the monkeys. However, these macaques serve as hosts for a large number of parasites, which are communicable to humans who are in contact with them. Therefore, their frequent contact with tourists possesses a potential health threat [14].

Gastrointestinal parasites may affect the health of macaques directly or indirectly, thereby exposing primates to conservational risks [15]. Numerous studies have identified, isolated and reported various gastrointestinal parasites in non-human primates [16,17,18]. The infection of intestinal parasites in rhesus monkeys kept in scenic spots affects the health of monkeys, as well as tourists and other domestic animals who visit those tourist sites [19,20]. Many studies have reported cases of human infection caused by intestinal parasites after contact with animals kept in the zoo, wildlife centers and ecological scenic areas in Africa, Southeast Asia and Southern China [21,22,23]. Wild or captive animals are significant in the epidemiology of various zoonotic diseases [24]. Gastrointestinal parasites cause various health conditions such as apathy, diarrhea, malaise and weight loss. However, there is still a dearth of knowledge about the population of intestinal parasites in macaques kept in the Mount Emei scenic spot. Parasitological studies are vital to appreciate the life cycle of parasites and the potential transmission route to other animals and humans [25]. Therefore, to evaluate and manage the effect of gastrointestinal parasites on any animal population dynamics, it is essential to evaluate their prevalence in wildlife populations [26]. The present study was undertaken to examine the status of gastrointestinal helminth and protozoan parasites in macaques at the Mount Emei scenic spot to help in designing future road maps in parasitic disease monitoring and control in wildlife habitats, as well as provide epidemiological data on parasites in monkeys, for education on public health and disease prevention.

## 2. Materials and Methods

### 2.1. Study Areas and Examined Monkeys

**Figure 1:**
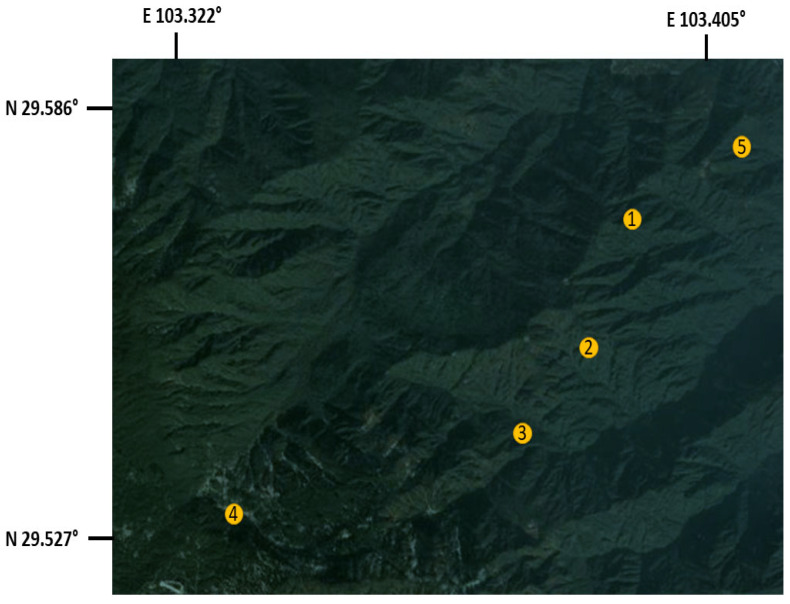
This map shows the summary of the study area (Emei Mountain scenic spot) and the geographical distribution of macaques: (1) yellow macaques group; (2) black macaques group; (3) new macaques group; (4) Leidongping macaques group; (5) Wuxiangang macaques group.

This study was carried out in the southwestern part of China (Figure 1). The study period was from 15 March 2019 (Spring season) to 16 January 2020 (Winter season). A total of 168 fecal samples were collected from seemingly healthy macaques (1–20 years old) of both sexes in the ecological area of the Emei Mountain scenic spot in the Sichuan province. The macaques had no anthelmintic treatment in the previous years, and these macaques had no direct contact with other monkeys and livestock. The macaques from the Mount Emei scenic spot were recorded and grouped according to their geographical location and special characteristics:

(1) Yellow macaques group (*n* = 31): the range of their activity was from Qingyinge to the ecological area. The group was characterized by yellow hair color and had more young monkeys in the population.
(2) Black macaques group (*n* = 19): the range of their activity was from near the trestle and cable bridge in the ecological area. The group was characterized by their dark brown color.
(3) New macaques group (*n* = 57): the range of their activity was from the ecological area to the upper section of Hongchunping. These macaques migrated to the ecological area near Jiulaodong in hongchunping. Their population was characterized by light brown hair color.
(4) Leidongping macaques group (*n* = 57): the range of activity was from the Leishen temple to Jinding ropeway near Leidongping. Their hair color was mostly grayish brown.
(5) Wuxiangang macaques group (*n* = 4): the range of their activity was from the Wuxiangang station to Qingyin Pavilion. This group was characterized by their unwillingness to be in contact with tourists.

### 2.2. Sample Collection

A total of 168 fecal samples from the yellow (*n* = 31), black (*n* = 19), new (*n* = 57), Leidongping (*n* = 57) and Wuxiangang (*n* = 4) macaques in the ecological area were collected between the periods of 15 March to 1 April (spring), 25 August to 10 September (summer), 15 June to 1 July (Autumn) 2019 and 2 January to 16 January (winter) 2020 from the Emei Mountain scenic spot. After clinical examination, the monkeys were initially anesthetized with intramuscular 15 mg/kg ketamine and 0.5 mg/kg acepromazine (HangZhou Testsea biotechnology Co., Ltd. Hangzhou, China), and fecal samples were aseptically collected directly from the rectum by disposable rectal gloves, placed into plastic bags and stored in polystyrene foam containers with recyclable ice, maintaining a temperature of about 4 °C within the container. The containers were labeled and then transferred to the Department of Clinical Veterinary Medicine, Sichuan Agricultural University for parasitological examination.

The occurrence time, location, contact times (including group photos, direct contact behaviors such as agonistic behavior of macaques and indirect contact behaviors including feeding and snatching) and the contact frequency between tourists and macaques (contact times/haunting time) of each of the monkey group were recorded daily.

### 2.3. Microscopic Examination of Fecal Parasites

Fecal samples were microscopically examined for helminth eggs and larvae following the method described previously [2].

### 2.4. Calculation of Prevalence Rate

Prevalence rate was calculated according to the formula by Thanasuwan et al. [27].

Prevalence (%) = number of macaques with parasites/total number of macaque × 100.

### 2.5. Statistical Analysis

All the data obtained were analyzed using SPSS 21.0 statistical software program. The chi-square test was used to determine the statistical significance of the different prevalence of endoparasites between macaque groups and seasons, respectively. Pearson’s correlation test was used to determine the relationship between the contact frequency and the intestinal parasite prevalence rate in the various macaque groups and seasons. In all the analyses, the confidence level was 95%, and statistical analyses were considered significant if *p* < 0.05 and highly significant if *p* < 0.01.

## 3. Results

### 3.1. Population and Daily Contact Frequency of Macaque in Various Seasons

The sampling time of each season was controlled within 10~20 days. The contact time between the macaques and tourists was recorded daily (including direct contact behaviors such as group photos, agonistic behaviors and indirect contact behaviors such as feeding and snatching). The daily contact frequency of the tourists was also calculated. See Table 1 and Table 2 for details.

### 3.2. Microscopic Examination Results and Analysis

**Figure 2:**
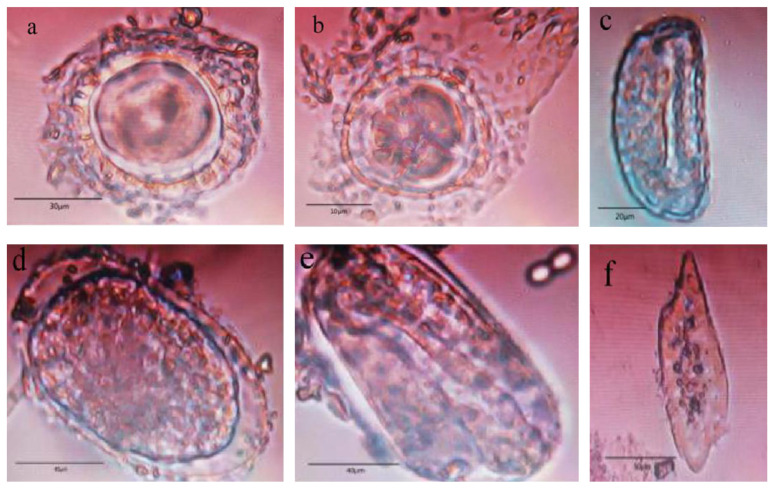
Gastrointestinal parasite presents in fecal samples of macaques. (**a**) Ascaris eggs; (**b**) trematoda eggs; (**c**) enterobius eggs; (**d**) physaloptera eggs; (**e**) ancylostoma duodenale eggs spp.; (**f**) cestoda egg.

A total of 168 fecal samples of the five formed groups of macaques were examined, and a total of 11 parasites were detected: 4 protozoa spp.—*Sphaerozoum fuscum*, *Balantidium coli*, *Entamoeba*, Blastocysts spp.—and 7 helminths spp.—Trematoda spp., *Gongylonema* spp., Cestoda, *Ascaris lumbricoides*, *Enterobius vermicularis*, *Ancylostoma duodenale* and *Physaloptera*. The total infection rate of the parasites was 51.19%. Among the helminth, *Gongylonema* spp. was highly detected in the fecal samples with a prevalence rate of 26.79% followed by Cestoda (4.17%), *Ancylostoma duodenale* (3.57%), Trematoda spp. (2.38%), *Physaloptera* spp. (1.79%), *Ascaris lumbricoides* (1.19%) and *Enterobius vermicularis* (0.60%). Moreover, among the protozoa, *Entamoeba* spp. had the highest prevalence rate (18.45%) followed by *Sphaerozoum fuscum* (14.29%), *Blastocysistis* spp. (2.98%) and *Balantidium coli* (1.19%). The species and prevalence rates of the intestinal parasites in the five macaque groups in the Mount Emei area are shown in Table 3 and Figure 2.

### 3.3. The Prevalence of the Intestinal Parasite in the Macaque Groups

**Figure 3:**
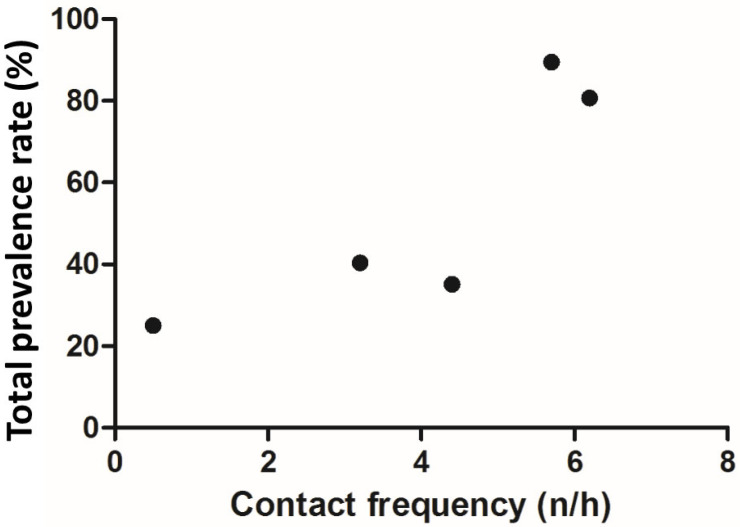
Correlation between the contact frequency and total intestinal parasite prevalence rate.

The prevalence rate of the intestinal parasite in the macaque groups is represented in Table 3. The prevalence rate of the intestinal parasite was arranged in the descending order of prevalence as follows; black macaque 89.47% (17/19), yellow macaque 80.64% (25/31), leidongping macaque 40.35% (23/57), new macaque 35.09% (20/57) and wuxiangang 25% (1/4). It was also observed that the black macaque group recorded the highest rate of intestinal parasite prevalence as compared to the other macaque groups (*p* < 0.05). In addition, we observed no significant difference in the rate of intestinal parasite prevalence among the yellow, leidongping and wuxiangang macaque groups (*p* > 0.05, *p* > 0.01). We also observed that the total intestinal prevalence rate significantly correlated with the contact frequency of tourists (r = 0.8479, *p* = 0.0348) (Figure 3).

### 3.4. The Prevalence of the Intestinal Parasite during Different Seasons

**Figure 4:**
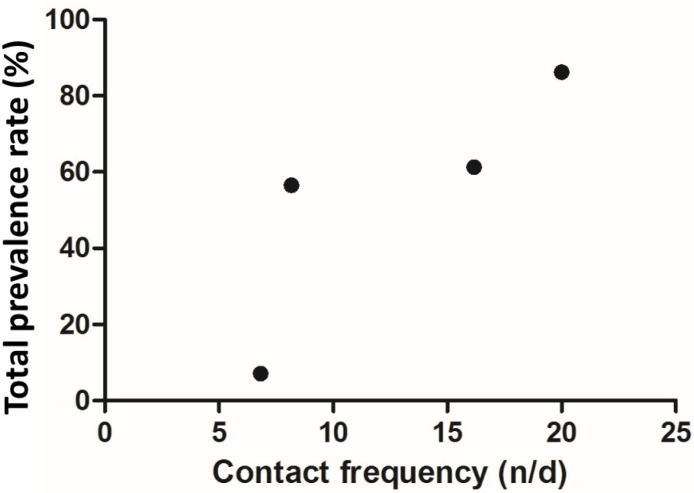
Correlation between the contact frequency and seasonal intestinal parasite prevalence.

Table 4 shows the results of the rate of prevalence of intestinal parasites in macaques in different seasons. The rate of prevalence was arranged in descending order of prevalence as follows: summer 86.21% (50/58), spring 61.29% (19/31), autumn 56.52% (13/23) and winter 7.14% (4/56). We observed that the summer season had the highest rate of prevalence of intestinal parasites compared with the other seasons (*p* < 0.05). Moreover, we also observed no significant correlation between the contact frequency and the prevalence rate of macaques during different seasons (r = 0.8349, *p* = 0.0825) (Figure 4).

## 4. Discussion

Mount Emei is a famous tourist attraction with a dense population flow and a spot where macaques have more contact with tourists. Humans and pets visiting scenic spots carry pathogens [28] and may transmit these pathogens when they are in direct or indirect contact with an animal in these scenic spots [29].

Among the intestinal parasites detected in the fecal samples of the macaques in the Mount Emei scenic area, *Gongylonema* spp., *Entamoeba* spp., *Sphaerozoum fuscum,* Cestoda spp. and *Ascaris lumbricoides* were the most dominant intestinal parasites, probably due to poor hygienic environment in the scenic area, untreated drinking water and free monkey contact with tourists. These observations were similar to the previous report [30]. In addition, we observed that the rate of prevalence of the intestinal parasites among the various groups of macaques was different, and thus we speculated that these differences could be caused by the difference in the geographical locations of the macaques in the scenic area, which was consistent with reports by Antonelli et al. [31]. However, there is a need for further studies.

*Gongylonema* spp., especially *Gongylonema pulchrum* (gullet worm), has been identified in many mammals [32,33]. Numerous cases of *Gongylonema* spp. infection with associated pathological lesions and clinical signs have been reported in monkeys and other vertebrates [34].

*Entamoeba* [35] is the species of amoeba that causes human infection and pathogenicity. They are free-living amoebas in water, air and soil, which were reported in the past as posing no threat to humans. However, few records of human infection with histolytic amoeba have been found in recent years [36], but their infection routes were not from their contact with other primates but mostly from water and soil source infections [37]. *Entamoeba histolytica* of *Entamoeba* spp. causes amoeba dysentery and liver abscesses [38]. *E. histolytica* trophozoites degrade the mucus layer, lyse epithelial cells and invade leukocytes [35,39]. *E. histolytica* also alters active electrolyte transport, secretion and malabsorption [40].

Adult tapeworms (Cestoda) are found in the small intestine. Cestoda infections are usually asymptomatic but may cause abdominal distress, dyspepsia, anorexia (or increased appetite), nausea, localized pain and diarrhea [41].

*Sphaerozoum fuscum* is a common and harmful parasite found in the intestine of most animals. It causes conditions such as emaciation, anemia, dysentery and growth inhibition [42].

*Ancylostoma duodenale* is the most common parasitic infection in countries with poor access to adequate water, sanitation and hygiene [43]. *Ancylostoma duodenale* has been reported to cause upper gastrointestinal bleeding [44]. Hookworms may cause Löffler syndrome with coughing, wheezing, eosinophilia and sometimes hemoptysis [45].

*Ascaris lumbricoides* is a common nematode parasite in humans and has been associated with intestinal pathology, respiratory symptoms and malnutrition in children from endemic areas [46]. *Ascaris lumbricoides* infects about 820 million people and is prevalent in at least 103 of 218 countries worldwide [47,48].

Numerous studies have reported that seasons can influence the growth and development of various endo- and ecto-parasites [31,49]. Thus, low temperatures affect the rate of growth, development and metabolism of parasites [50]. A study by Viljoen et al. [51] showed that clear seasonal patterns of parasite prevalence and abundance emerged with peaks during the summer season for mites and the winter season for cestodes. In the present study, the highest prevalence rate was recorded during the summer season, and this may be related to many helminths developing during the summer season [52].

Contact between humans and animals is unavoidable [53], which may result in the natural transmission of parasites and zoonotic diseases [54]. Therefore, in this study, Pearson correlation analysis was used to determine whether there was a possible relationship between the frequency of human contact and the prevalence rate of the intestinal parasites, as well as the frequency of seasonal human contact and the seasonal prevalence rate of the intestinal parasites. The results showed a positive correlation between the prevalence rate of the intestinal parasites and human contact with the monkeys, indicating a high probability of monkeys transmitting these intestinal parasites to tourists (vice versa). This finding was inconsistent with the previous study by Elbahy et al., [52]. However, there was no significant correlation between the seasonal prevalence rate of the parasites and seasonal human contact with monkeys.

## 5. Conclusions

The presence of gastrointestinal helminth and protozoa parasites with known ill impact on macaques may threaten the survival of these monkeys, as well as tourists and other animals living in the Mount Emei scenic area. Based on our parasitological results, we found various gastrointestinal endoparasites in the macaques in the Mount Emei scenic area. Among them, *Gongylonema* spp. and Entamoeba were the prevalent parasites, whereas *Gongylonema* spp., Entamoeba spp., *Ascaris lumbricoides*, *Ancylostoma duodenale* and Cestoda were the dominant zoonotic parasites in the gastrointestinal tract of the macaques. Therefore, restrictions to reduce direct human (tourists) contact with macaques, proper facility management, keeping good sanitary conditions of the scenic area and regular deworming of monkeys are recommended. In addition, we suggest further research to be conducted to determine the intensity of infection, parasitic diseases and mode of transmission of parasites and possible mitigating strategies to strengthen the management of the scenic area.

## Figures and Tables

**Table 1:** Population of monkeys during the four seasons.

Population Season	Yellow Macaque Group in the Ecological Area	Black Macaque Group in the Ecological Area	New Macaque in the Ecological Area	Leidongping Macaque	Wuxiangang Macaque	Total
Spring	9	4	7	9	2	31
Summer	14	9	16	17	2	58
Autumn	5	3	6	9	0	23
Winter	3	3	28	22	0	56
Total	31	19	57	57	4	168

**Table 2:** Contact frequency of different groups of macaques with humans.

Monkeys	Number of Samples	Haunt Time Every Day (h)	Haunt Location	Daily Exposure Time (*n*)	Contact Frequency (*n*/h)
Yellow macaque	31	2.6	Near Qingyin Pavilion	16.1	6.2
Black macaque	19	2.9	Suspension bridge in ecological area	16.4	5.7
New macaque	57	3.2	Sandaoqiao, suspension bridge in ecological area	14.1	4.4
Leidongping macaque	57	6.1	Leidongping stand	19.5	3.2
Wuxiangang macaque	4	0.6	Wuxiangang station	0.3	0.5

**Table 3:** Total prevalence rate of the intestinal parasites in macaques.

Population	Yellow Macaque Group in the Ecological Area (*n* = 31)		Black Macaque Group in the Ecological Area (*n* = 19)		New Macaque in the Ecological Area (*n* = 57)		Leidongping Macaque (*n* = 57)		Wuxiangang Macaque (*n* = 4)		Total (*n* = 168)	
Parasites	DN (*n*)	PR (%)	DN (*n*)	PR (%)	DN (*n*)	PR (%)	DN (*n*)	PR (%)	DN (*n*)	PR (%)	DN (*n*)	PR (%)
*Ascaris lumbricoides*	2	6.45	0	0	2	3.51	0	0	0	0	2	1.19
*Entamoeba* spp.	12	38.71	7	36.84	5	8.77	7	12.28	0	0	31	18.45
*Blastocysistis* spp.	0	0	1	5.26	3	5.26	0	0	0	0	5	2.98
*Enterobius vermicularis*	0	0	0	0	0	0	1	1.75	0	0	1	0.60
Trematoda spp.	0	0	1	5.26	2	3.51	1	1.75	0	0	4	2.38
Cestoda spp.	5	16.13	0	0	1	1.75	2	3.51	0	0	7	4.17
*Gongylonema* spp.	13	41.94	12	63.16	6	10.56	13	22.81	1	25	45	26.79
*Ancylostoma duodenale*	0	0	1	5.26	3	5.26	2	3.51	0	0	6	3.57
*Physaloptera* spp.	0	0	0	0	1	1.75	2	3.51	0	0	3	1.79
*Balantidium coli*	1	3.23	1	5.26	0	0	0	0	0	0	2	1.19
*Sphaerozoum fuscum*	8	25.81	1	5.26	7	12.28	5	8.77	1	25	24	14.29
Total	25	80.65	17	89.47	20	35.09	23	40.35	1	25	86	51.19

DN—Detection number, PR—Prevalence rate.

**Table 4:** Prevalence of intestinal parasites during different seasons.

Season/Population Parasites	Spring (*n* = 31)		Summer (*n* = 58)		Autumn (*n* = 23)		Winter (*n* = 56)		Total (*n* = 168)	
	DN (*n*)	PR (%)	DN (*n*)	PR (%)	DN (*n*)	PR (*n*)	DN (*n*)	PR (%)	DN (*n*)	PR (%)
*Ascaris lumbricoides*	1	3.23	1	1.72	1	4.35	1	1.79	4	2.38
*Entamoeba* spp.	0	0	31	53.45	0	0	0	0	31	18.45
*Blastocysistis* spp.	0	0	4	6.90	0	0	0	0	4	2.38
*Enterobius vermicularis*	0	0	0	0	1	4.35	0	0	1	0.60
Trematoda spp.	0	0	1	1.72	3	13.04	0	0	4	4.76
Cestoda spp.	1	3.23	4	6.90	3	13.04	0	0	8	4.17
*Gongylonema* spp.	15	48.38	18	31.03	9	39.13	3	5.36	45	26.79
*Ancylostoma duodenale*	0	0	5	8.62	1	4.35	0	0	6	3.57
*Physaloptera* spp.	0	0	3	5.17	0	0	0	0	3	1.79
*Balantidium coli*	1	3.23	1	1.72	0	0	0	0	2	1.19
*Sphaerozoum fuscum*	4	12.90	16	27.59	2	8.70	0	0	22	13.10
Total	19	61.29	50	86.21	13	56.52	4	7.14	86	51.19

DN—Detection number, PR—prevalence rate.

## Data Availability

The data presented in this study are available on request from the corresponding author.
